# Investigation of Coked Catalyst Regeneration via Non-Thermal Plasma Treatment and Its Reuse for Hydrogen Production from Methane Pyrolysis

**DOI:** 10.3390/molecules31101733

**Published:** 2026-05-19

**Authors:** Šarūnas Varnagiris, Marius Urbonavičius, Simona Tučkutė, Vishnu Radhakrishnan Nair, Ainars Knoks, Liga Grinberga, Raitis Kaspars Sika, Brigita Kmet, Danjela Kuscer

**Affiliations:** 1 Center for Hydrogen Energy Technologies, Lithuanian Energy Institute, LT-44403 Kaunas, Lithuania; marius.urbonavicius@lei.lt (M.U.); simona.tuckute@lei.lt (S.T.); vishnu.nair@lei.lt (V.R.N.); 2 Institute of Solid State Physics, University of Latvia, LV-1063 Riga, Latvia; ainars.knoks@cfi.lu.lv (A.K.); liga.grinberga@cfi.lu.lv (L.G.); raitis.sika@cfi.lu.lv (R.K.S.); 3 Electronic Ceramics Department, Jožef Stefan Institute, 1000 Ljubljana, Slovenia; brigita.kmet@ijs.si (B.K.); danjela.kuscer@ijs.si (D.K.)

**Keywords:** methane pyrolysis, hydrogen, Fe-based catalyst, coked catalyst, deactivation, non-thermal plasma treatment, catalyst regeneration

## Abstract

As a low-carbon alternative, methane pyrolysis offers a viable approach to overcoming the emission challenges associated with traditional hydrogen generation. However, catalyst deactivation is one of the key challenges, mainly caused by high-temperature sintering and coke deposition that block active sites. This study investigates the application of non-thermal plasma (NTP) treatment for the regeneration of coked catalysts through in situ carbon removal and performance recovery. Carbon removal by NTP is proposed as a cleaner alternative to conventional regeneration methods. The influence of plasma treatment was evaluated under different plasma treatment configurations, including the use of an auxiliary magnet to direct plasma flux toward the targeted region, and variations in gas composition (H_2_/Ar and H_2_/O_2_). The plasma-treated catalyst was analyzed by SEM, EDS, XPS, and XRD techniques. Additionally, samples were evaluated for hydrogen production via methane pyrolysis. The results demonstrated measurable surface carbon removal, reaching approximately 38%. However, methane pyrolysis experiments revealed that this level of surface carbon removal was insufficient to achieve substantial catalytic activity recovery, indicating the need for further optimization.

## 1. Introduction

Clean and sustainable energy is critical for meeting rising global energy demands while reducing environmental impacts. In this context, hydrogen has attracted increasing attention as a versatile energy carrier rather than a primary energy source. Its significance lies in its ability to link different parts of the energy system, since it can be produced from multiple feedstocks, stored, transported, and then converted into electricity, heat, or chemicals depending on demand [1]. At the system level, hydrogen is considered particularly relevant where direct electrification is difficult, including hard-to-abate industrial sectors, chemical reducing environments, and long-duration or seasonal energy storage. However, its deployment also depends strongly on the availability of production, transport, storage, and distribution infrastructure, which remains a major constraint for large-scale adoption [2]. These broader system and supply chain challenges have been emphasized in recent reviews of hydrogen production and supply chain development [1,3].

Hydrogen already plays an essential role in petroleum refining and ammonia synthesis, and it is also being considered for fuel cells, power generation, and grid-scale energy storage [4,5,6]. Its main advantages include high gravimetric energy content (~120 MJ kg^−1^), fuel flexibility, and the possibility of low direct emissions at the point of use, especially in fuel-cell-based systems [7]. At the same time, hydrogen also faces important limitations when compared with other energy options: it has low volumetric energy density, requires energy-intensive storage and transport, and its environmental benefit depends strongly on how it is produced [8]. As discussed in the hydrogen energy and storage literature, these advantages and limitations must be considered together when evaluating hydrogen against alternative energy pathways.

At present, most hydrogen is produced from fossil resources, primarily via steam methane reforming (SMR) and coal gasification, which substantially limits its climate benefit because these routes are carbon-intensive and typically emit approximately 9–12 kg of CO_2_ per kilogram of H_2_ produced [9]. Therefore, the development of efficient and cleaner hydrogen production technologies remains a central scientific and technological priority [10]. Beyond reforming-based routes, several modern approaches are being actively explored. Photocatalytic hydrogen production uses light-excited semiconductors to drive water splitting or related hydrogen-evolution reactions; its main attractions are the possibility of solar-driven operation and the use of earth-abundant, non-noble-metal systems, but low conversion efficiency, charge-recombination losses, and limited scalability remain current limitations [11,12]. Water electrolysis generates hydrogen by splitting water with electricity and is currently the most mature low-emission route when coupled with renewable power, although its large-scale deployment is still constrained by electrolyzer cost, durability and the high electricity demand required for competitive operation [13,14]. Formic acid decomposition releases hydrogen catalytically from a liquid hydrogen carrier under comparatively mild conditions, which is attractive for on-demand hydrogen delivery, but it is better viewed as a hydrogen storage and release pathway than as a primary production route and catalyst selectivity is critical because CO formation is highly undesirable [15]. Ammonia decomposition converts NH_3_ into H_2_ and N_2_ and benefits from ammonia’s high hydrogen density and established storage and transport infrastructure, but efficient conversion still requires highly active catalysts and adequate control of hydrogen purity and reaction conditions [16,17].

Among these alternatives, methane pyrolysis has attracted increasing interest as an endothermic one-step process that decomposes methane into hydrogen and solid carbon, thereby avoiding direct CO_2_ formation in the main reaction step [18,19]. Carbon may also represent a valuable co-product in the process value chain [20]. In this respect, methane pyrolysis can be viewed as an alternative to conventional fossil-based hydrogen production routes that form gaseous carbon oxides and require subsequent carbon management [21]. Compared with electrolysis, methane pyrolysis can reduce dependence on large electricity inputs, while compared with formic acid or ammonia decomposition, it directly generates hydrogen from a primary feedstock rather than from a previously synthesized hydrogen carrier [13,16]. Compared with photocatalytic routes, it is also closer to thermochemical reactor-scale implementation. At the same time, these advantages are conditional on system-level factors, including the carbon footprint of the methane source, the energy required to drive methane activation, and the effective handling and utilization of the solid carbon produced [22].

Methane pyrolysis has also been identified as a potentially energy-efficient hydrogen production route compared with processes such as SMR or water electrolysis [21,22]. In addition, methane pyrolysis has been reported to require a lower specific energy input than several conventional hydrogen production routes, with representative values of 37.8 kJ mol^−1^ H_2_ compared with 63.3 kJ mol^−1^ H_2_ for steam reforming and 285.9 kJ mol^−1^ H_2_ for water electrolysis [23]. However, the high thermodynamic stability of methane means that breaking its strong C–H bonds still requires substantial energy input and effective catalytic assistance. Catalysts are therefore essential not only for accelerating methane conversion and lowering activation barriers, but also for influencing the morphology and nature of the carbon byproducts formed [24].

A major limitation in catalytic methane pyrolysis is catalyst deactivation. This occurs primarily through metal particle sintering at high temperatures and the formation of carbonaceous deposits (coke) on active sites. Such deactivation is often driven by an imbalance between the methane conversion rate and the carbon diffusion rate on the catalyst surface, resulting in excessive carbon accumulation [25,26]. Over time, catalytic metal crystallites become isolated and encapsulated in carbon filaments, ultimately leading to total catalyst deactivation. Understanding these mechanisms is critical for developing catalyst regeneration methods, particularly Fe and Ni-based systems, which are commonly used due to their high activity but are highly susceptible to coke formation [27].

Catalyst regeneration is vital to sustaining the performance and economic feasibility of MP. Traditional regeneration techniques used to remove coke from the spent catalysts include combustion with oxygen (air regeneration) [28,29], steam gasification [30], and, to a lesser extent, CO_2_ gasification [31]. In air regeneration, oxygen burns the accumulated coke, producing CO_2_ (or CO if incomplete is combustion) [32]. Although this restores catalyst activity, it often reduces selectivity toward valuable carbon nanotube byproducts [33] and may lower the reducibility of nickel particles [34]. Steam regeneration, being endothermic, involves two stages: the initial production of pure hydrogen, followed by a stage that introduces CO and CO_2_ impurities [29]. This method has shown improved catalyst stability, with Ni/SiO_2_ systems maintaining activity over regeneration cycles at 650 °C [35]. CO_2_ gasification, in which CO_2_ reacts with coke to form CO, has also been explored but remains limited due to its high energy requirement, low carbon removal rate, and tendency to cause catalyst agglomeration and deactivation, particularly using TiO_2_, SiO_2_, and Al_2_O_3_ as support [34].

Alternative chemical methods, such as nitric acid refluxing, have also been explored. Wang et al. reported recovery of CNTs with 96% purity from Fe/Al_2_O_3_ catalysts after acid treatment [24]. More recently, Alves et al. introduced an interfacial hydrogenation approach involving cyclic H_2_ refeeding, which successfully regenerated bulk Ni catalysts after 22 MP cycles at 550 °C and 1 bar [36]. Similarly, Ni–CeO_2_-based nanoparticle clusters have been regenerated via chemical looping using the reverse Boudouard reaction [37]. These methods show promise for improving catalyst stability and performance, but issues remain regarding CO_2_ emissions, chemical and solvent use, high energy demands, economic viability, scalability, and process integration complexity.

Plasma-based techniques have attracted increasing interest in catalyst regeneration, particularly for their ability to selectively remove coke without damaging the catalyst structure. Non-thermal plasma (NTP) operates at low bulk gas temperatures while maintaining high electron energies (1–10 eV), enabling coke removal without thermal degradation [38]. NTP interacts with coke via multiple pathways: electron-impact dissociation, radical and ozone formation, vibrational excitation, and physical sputtering [39,40]. The pioneering work of Bibby et al. demonstrated coke removal from ZSM-5 zeolites using radio frequency oxygen plasma, achieving 94% removal within 15 min through reactions between coke deposits and plasma-generated atomic oxygen [41,42]. In later work, the same authors observed improved coke elimination when using Ar–O_2_ mixtures [43,44]. Dielectric barrier discharge (DBD) plasma systems have since demonstrated coke removal rates of 70–94% with less sintering effect than radiofrequency plasmas [40,45]. DBD systems also operated at lower power and achieved faster cleaning than traditional AC sine-wave signals, as shown in decoking noble metal catalysts (Pt–Sn/Al_2_O_3_ and Au/TiO_2_) [46,47].

In this study, we investigate the regeneration of Fe-based catalysts coked during methane pyrolysis using a novel NTP system enabled by plasma electrodes, operating under inert and reactive gas atmospheres. Unlike most studies focused primarily on catalyst synthesis or catalytic performance screening, the present work addresses the less explored but technologically critical problem of post-reaction catalyst regeneration and reuse. To our knowledge, this is the first application of such a system for coke removal in Fe-based catalysts. Plasma treatment parameters were systematically examined, including gas composition (H_2_/Ar and H_2_/O_2_), input power, operating pressure, exposure time, and the use of an auxiliary magnet to direct plasma flux toward the targeted region in order to achieve effective coke removal. The integration of magnetron-assisted plasma not only minimizes CO_2_ emissions but also facilitates catalyst reuse for the next MP cycle. The innovative aspect of this work therefore lies in introducing and assessing a plasma-assisted regeneration concept, including magnet-assisted plasma focusing, as a route to recover the functionality of coked catalysts while avoiding conventional oxidative regeneration pathways. In this way, this study contributes a regeneration-focused perspective to methane pyrolysis by examining how plasma treatment affects carbon removal, spent-catalyst surface state, and the potential for catalyst reuse.

## 2. Results and Discussion

### 2.1. Plasma Treatment

**Figure 1:**
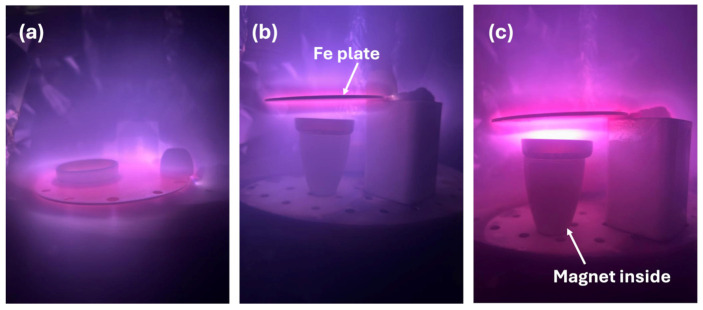
Plasma treatment modifications: Fe plate for glow discharge plasma and Petri dish with coked catalyst (**a**) placed on Fe plate; (**b**) beneath Fe plate; (**c**) beneath Fe plate with magnetic rod inside ceramic crucible.

In this work, the material recovered after methane pyrolysis is referred to as a coked catalyst, reflecting the formation of carbon deposits on the catalyst surface during pyrolysis. Non-thermal plasma treatment was applied to remove the deposited coke. The non-thermal plasma treatment experiments were conducted using an Fe target, where a glow-discharge plasma was initiated with pure H_2_ and H_2_/Ar or H_2_/O_2_ mixtures. Three NTP treatment configurations were tested to initiate carbon removal from the catalyst surface. In the first approach (Figure 1a), a glass Petri dish containing the coked catalyst was placed directly on the Fe plate. Various plasma treatment parameters were investigated, including gas composition, plasma power, pressure, and treatment duration. However, only a few percent of surface carbon removal were achieved in this configuration. The second approach involved positioning the glass Petri dish beneath the Fe plate on a ceramic crucible (Figure 1b) instead of placing it directly on top. This configuration resulted in a slight improvement in carbon removal efficiency; however, the overall removal remained low. The limited carbon removal was attributed to the concentration and weak interaction of generated plasma with the coked catalyst. Therefore, a third approach was designed to deliberately guide the plasma flux: the glass Petri dish was placed beneath the Fe plate on a ceramic crucible containing a magnetic rod (Figure 1c).

The presence of magnetic fields can significantly alter the shape and dynamics of glow discharge plasma by influencing charged particle trajectories and plasma confinement. External magnetic fields are known to modify plasma parameters, such as electron density and temperature, by confining electrons along magnetic field lines and redistributing the plasma spatially, thereby altering the effective plasma volume and discharge characteristics compared to magnet-free conditions [48].

This effect has been exploited in related plasma-processing applications, where magnetic fields are used to enhance plasma confinement and extend the discharge region, effectively shaping the plasma for targeted surface interactions [49]. In our setup, the magnet embedded in the ceramic crucible likely perturbs the local magnetic field distribution, thereby distorting the glow plasma shape and enhancing interaction with the coked catalyst surface. This magnetically assisted configuration was therefore employed in all subsequent plasma-treatment experiments.

### 2.2. Structural and Chemical Analysis

Table 1 and Figure S1 in the Supplementary Materials present the changes in the surface elemental concentration of the coked catalyst before and after plasma treatment under different gas compositions. Because XPS is a surface-sensitive technique probing only the outer few nanometers of the material, the measured carbon values should be interpreted as changes in surface carbon atomic concentration rather than as the total amount of carbon present in the porous Fe–Ni/Al_2_O_3_ catalyst. In such porous materials, carbon deposits may also be located inside pores and around embedded metal particles, and these contributions are not fully captured by XPS. Therefore, the carbon removal values discussed below reflect primarily surface carbon removal induced by plasma treatment.

The surface of the coked catalyst after methane pyrolysis consisted of carbon (77.4 at. %), oxygen (15.4 at. %), and aluminum (7.2 at. %). This indicates that the external surface was heavily covered by carbonaceous deposits, while the catalyst substrate (Al_2_O_3_) was only weakly exposed. Fe and Ni were not detected on the coked surface under these conditions. This coked catalyst was treated under various plasma concentrations to initiate its regeneration (surface carbon removal), including pure H_2_, and various H_2_ mixtures with Ar (from 3% to 20%), and O_2_ (from 3% to 20%). The results revealed that pure H_2_ gas is insubstantial for catalyst regeneration, resulting in only a 1.5 percent reduction in carbon content (from 77.4 at. % to 76.2 at. %). Although hydrogen plasma is chemically reactive, when used alone, its effectiveness for removing carbon films or cleaning surfaces is often limited due to the extremely low mass of hydrogen atoms [50]. Therefore, hydrogen is commonly combined with heavier gases such as argon to enhance sputtering, or with oxygen to enable efficient oxidative removal pathways, thereby significantly improving surface carbon removal compared to pure hydrogen plasma [51,52].

**Figure 2:**
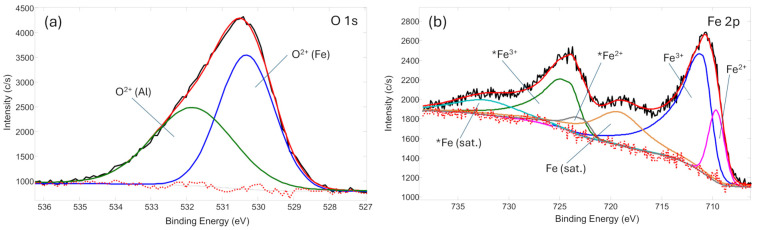
XPS chemical bond analysis of (**a**) O 1s, and (**b**) Fe 2p spectra after plasma treatment at H_2_ (95%) + O_2_ (5%) gas mixture. Peaks marked with “*” correspond to Fe 2p_1/2_ components, whereas unmarked peaks correspond to Fe 2p_3/2_ components. Black line: measured spectra; red line: fitted spectra; red dotted line: background; other colored lines: deconvoluted spectral components.

In subsequent experiments, gas mixtures of H_2_ with Ar and with O_2_ were investigated. The results showed that increasing the Ar content from 3% to 20% increased surface carbon removal from 7.8% to 33.2%. In comparison, the use of H_2_-O_2_ mixtures resulted in higher surface carbon removal, increasing from 12.5% to 38.6% as the O_2_ concentration was raised from 3% to 20%. At the same time, an increase in oxygen concentration was observed in both types of gas mixtures from 15.4% in the coked catalyst to 35.2% and 37.7% after 20% Ar and 20% O_2_ mixtures with H_2_, respectively. This is mainly related to the carbon removal procedure, by opening the catalyst substrate (Al_2_O_3_), while the greater increase in oxygen in the H_2_-O_2_ plasma treatment case is possibly related to the formation of oxide-based compounds on top of the catalyst. This was further confirmed by XPS analysis, by deconvoluting Fe-O chemical bonds (Figure 2). It is noteworthy that the carbon removal process initiated the increase in both Al and Fe chemical concentrations. XPS was used in this work because the aim of this study was to assess plasma-induced surface modification in the catalyst region directly exposed to the discharge, including changes in surface carbon concentration, surface oxidation, and Fe chemical state after treatment. This makes XPS appropriate for tracking surface-level effects of plasma exposure. However, for a porous catalyst, residual carbon located within pores or less accessible internal regions might remain undetected by this method. This limitation is also important for interpreting the catalytic results, because incomplete recovery of methane pyrolysis performance may be associated with the persistence of coke in pore-confined or subsurface regions not probed by XPS.

Additionally, to clearly understand the side effects associated with plasma treatment, an additional set of experiments was performed on quartz plates instead of the catalyst. A pure quartz plate was used to investigate potential Fe deposition during the plasma treatment process. All plasma treatment conditions were kept identical to those used for catalyst plasma treatment, as described in Section 3.3, “Coke Removal via Plasma Treatment”. It was observed that Fe was also deposited during plasma treatment. The amount of deposited Fe depends on the Ar or O_2_ gas in H_2_. Measurements of Fe thickness on quartz showed that using a very low Ar or O_2_ concentration (3%) results in less than 50 nm deposition of Fe in both cases. However, by increasing the amount of Ar or O_2_ gas up to 20% in H_2_ gas, the amount of deposited Fe increases to 300 nm and 160 nm, respectively. The higher Fe deposition rate observed with the H_2_-Ar gas mixture compared to the H_2_-O_2_ gas mixture is reasonable due to the higher sputtering yield associated with the heavier, inert Ar^+^ ions and the absence of target poisoning effects, whereas reactive oxygen leads to oxide formation on the Fe target and reduces the efficiency of momentum transfer during sputtering [53].

These results show that the plasma treatment is not a purely decoking process because it may also introduce Fe re-deposition onto the treated surface. Since Fe is an active phase for methane pyrolysis, this effect complicates the interpretation of post-treatment catalytic performance. The quartz measurements provide evidence for the tendency of Fe re-deposition during plasma treatment, but they do not directly quantify how much Fe is deposited on the porous catalyst itself or how that Fe is distributed over the catalyst surface and pore structure. Accordingly, the observed behavior after plasma treatment cannot be attributed solely to coke removal as it may reflect a combination of partial surface decoking, surface oxidation/restructuring and limited Fe re-deposition. In the present study, the plasma-treated catalyst is therefore interpreted primarily as a surface-modified catalyst rather than as a fully and selectively regenerated one.

In this work, we mainly focus on investigating the most appropriate approach for surface carbon removal by plasma treatment, aiming to regenerate the catalyst by removing coke deposits and reopening existing active catalytic sites, rather than attempting catalyst regeneration through the deposition of new catalytic material onto an already coked surface. Therefore, subsequent investigations concentrate on catalysts treated in an H_2_ (95%) + O_2_ (5%) gas mixture during plasma exposure. Under these conditions, a surface carbon removal efficiency of 35.6% was achieved (carbon content reduced from 77.4% to 49.8%), accompanied by a relatively moderate Fe deposition of ~60 nm. This condition was therefore selected as the most suitable compromise for examining plasma-induced surface modification under conditions of lower Fe sputtering than those observed at higher Ar or O_2_ contents. Nevertheless, a catalytically relevant contribution from deposited Fe on the actual porous catalyst cannot be excluded or quantified from the present data. Accordingly, the results obtained after plasma treatment should be interpreted cautiously as reflecting combined effects of partial surface decoking and plasma-induced surface modification.

The chemical bond analysis was performed on the plasma-treated catalyst to understand its state, specifically the Fe catalytic material (Figure 2). Deconvolution of the O 1s peak revealed two oxygen chemical environments, assigned to O–Al and O–Fe species at binding energies of 530.3 eV and 531.8 eV, respectively. These components are attributed to the Al_2_O_3_ catalyst substrate and the oxidized catalyst surface. However, the O 1s spectrum is not suitable for distinguishing Fe oxidation states. Therefore, the Fe 2p region was evaluated. Analysis of the Fe 2p spectrum revealed the presence of oxidized iron species, with Fe^2+^ and Fe^3+^ components observed at binding energies of 709.6 eV and 711.0 eV, respectively, along with their corresponding Fe 2p_1/2_ components and associated shake-up satellites. Notably, no metallic Fe^0^ species were detected in the Fe 2p spectrum. These results indicate that, following catalyst regeneration, the exposed Fe-based catalytic sites are fully oxidized, despite the additional Fe deposition observed during plasma treatment. The prevalence of these oxidized species at the catalyst surface might be a significant factor in the initial catalytic behavior, where the fully oxidized state observed here can likely act as a kinetic barrier.

**Figure 3:**
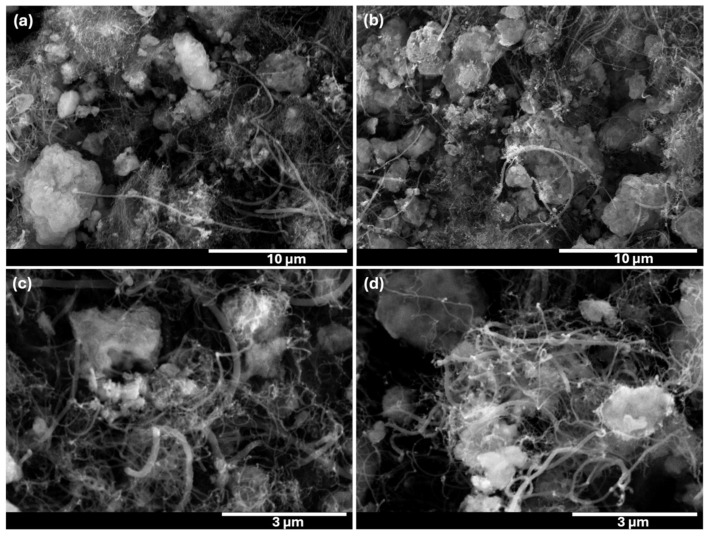
SEM images of (**a**,**c**) the coked catalyst before and (**b**,**d**) after plasma regeneration.

SEM analysis of the catalyst shows irregular, angular particle morphologies that remain characteristic of the material after methane pyrolysis and subsequent plasma treatment (Figure 3). In addition to the particle framework, carbonaceous filaments are observed on the surface after methane pyrolysis. These features are consistent with carbon nanofibers formed during methane decomposition on the catalyst, where carbon precipitates at active metal sites and grows in filamentous form. The filaments appear anchored to or extending from the catalyst particles, indicating catalytic filament growth rather than non-selective amorphous carbon deposition. Carbonaceous deposits are present as both filamentous structures and thin, conformal carbon layers, which are difficult to resolve on rough metallic surfaces. After regeneration the overall particle morphology remains unchanged, while filamentous features are still locally observed. This lack of pronounced morphological changes suggests that the H_2_-O_2_ plasma removes carbon predominantly through chemical etching, converting surface carbon into C-O based species, rather than through physical sputtering or surface reorganization [54]. The SEM observations, supported by XPS data, indicate that plasma regeneration primarily affects surface chemistry while preserving the underlying catalyst structure.

The elemental mapping of the catalyst shows the presence of carbon on the surfaces, both before and after plasma regeneration (Figure S2 in the Supplementary Materials). Prior to regeneration, the carbon signal appears relatively uniform and spatially correlated with the particle surfaces, consistent with the formation of carbonaceous deposits during pyrolysis. After plasma regeneration, the elemental maps still indicate the presence of carbon on the powder surfaces, and no pronounced qualitative change in the spatial distribution of carbon is observed. The similarity between pre- and post-regeneration maps reflects a known limitation of SEM-EDS mapping: elemental mapping alone does not provide sufficient sensitivity to assess changes in total carbon content reliably, and the persistence of a carbon signal in the maps does not necessarily imply ineffective regeneration. It is known that SEM-EDS mapping may overrepresent residual carbon due to strong surface sensitivity and limited depth resolution, whereas quantitative EDS spectra are more reliable for tracking relative compositional changes [55,56]. Therefore, the elemental analysis of the plasma-treated surface was performed by the XPS technique, which is much more suitable for top-layer catalyst elemental and chemical bond analysis.

**Figure 4:**
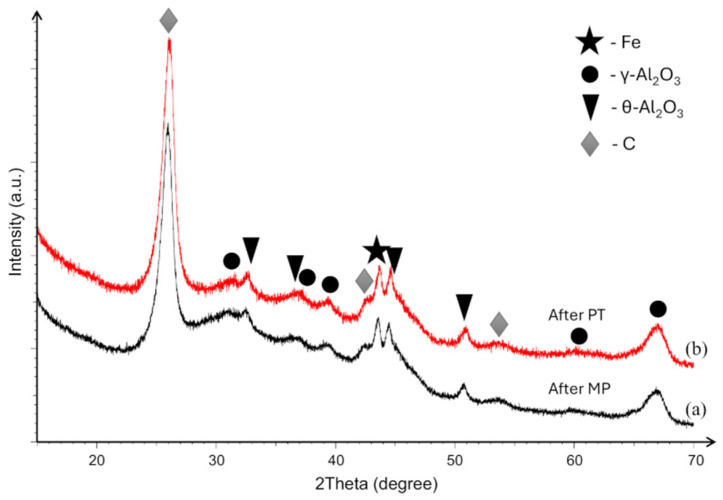
XRD patterns of the catalyst after (**a**) methane pyrolysis (after MP, black line) and (**b**) after plasma treatment (after PT, red line).

XRD patterns of the catalyst after methane pyrolysis (After MP) and after plasma treatment (After PT) are presented in Figure 4. In both cases, the diffraction spectra are dominated by a strong reflection at around 2θ ≈ 26°, corresponding to carbon formed during methane pyrolysis, confirming extensive carbon deposition on the catalyst surface. The analysis further shows that the support retains its characteristic cubic γ-Al_2_O_3_ phase. Additional peaks attributed to monoclinic θ-Al_2_O_3_ are also observed; the presence of this phase is most likely associated with a partial γ → θ transformation or localized thermal fluctuations occurring during MP and subsequent handling. A distinct diffraction peak associated with metallic Fe is detected near 2θ ≈ 45°, indicating the presence of crystalline iron species within the catalyst. In contrast, due to the nanoscale nature of the Ni nanoclusters, no distinct Ni-related diffraction peaks can be resolved. Their diffraction signals are likely masked by the intense reflections of the Al_2_O_3_ support and by the overlapping peak positions of Fe and Ni phases. While XRD analysis confirmed the presence of metallic Fe in the bulk, the XPS results indicate that the plasma treatment results in an oxidized surface layer (Figure 2b). This suggests the possible formation of a core–shell like structure, where a metallic iron core is encapsulated by an iron oxide passivating layer. Given that methane pyrolysis is a surface-sensitive reaction, the absence of surface Fe^0^ sites, despite their presence in the bulk, might negatively affect the catalytic performance.

Importantly, the XRD patterns before and after plasma treatment are highly similar, with no detectable phase changes and no significant reduction in the intensity of the carbon-related reflection. This demonstrates that the non-thermal plasma treatment does not alter the bulk crystallographic structure of the catalyst and that carbon removal under the present conditions remains limited mainly to the accessible surface region, consistent with the XPS and SEM findings. Overall, the XRD analysis confirms that plasma-assisted regeneration preserves the structural integrity of the catalyst while enabling coke removal at the surface level.

### 2.3. Methane Pyrolysis Using Regenerated Catalyst

**Figure 5:**
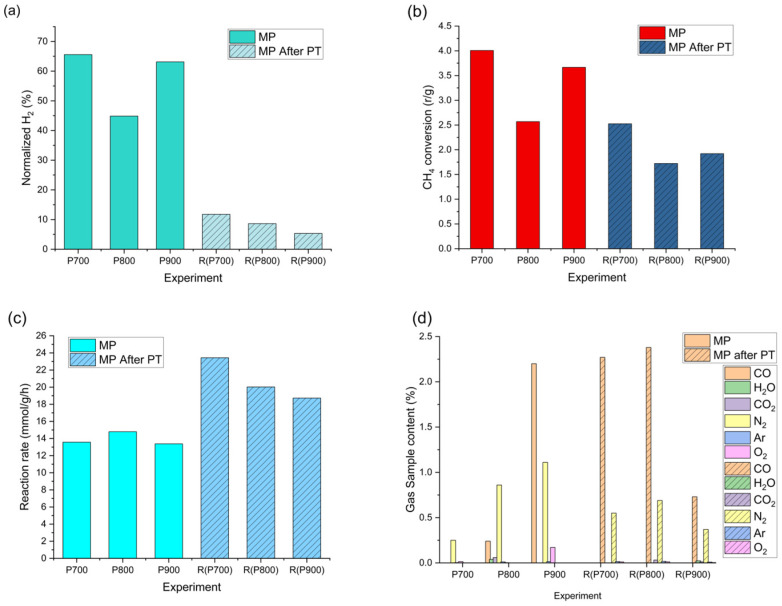
Gas content and reaction rate comparison of Methane pyrolysis, (**a**) gas content after MP, (**b**) CH_4_ conversion rate per gram of catalyst and (**c**) H_2_ production rate. Initial MP vs. after plasma treatment (MP after PT) and (**d**) Residual gas composition comparison.

Initial pristine MP was carried out at 700, 800, and 900 °C, with samples labeled, respectively, P700, P800, and P900. The methane pyrolysis experiments were performed for plasma-treated catalysts, and gas analysis was done to evaluate the produced H_2_ amount; for quantification, the amount of H_2_ and CH_4_ was normalized to 1 mol of CH_4_, and gas content was recalculated for carbon content in solid and gaseous forms. This allowed an easily comparable value, methane conversion rate, and hydrogen production rate to be depicted in Figure 5. The first MP results show around 60% H_2_ content, while after PT, the content decreases to around 12%, as shown in Figure 5a. It is noteworthy that due to specific reactor setup and current sample production limitations, as regards the absolute observed gas content after plasma treatment, the hydrogen content reaches 20% of the pristine sample. Moreover, samples with increased initial MP reaction temperature yield a decrease in the amount of hydrogen in gas sample in second MP reaction. For quantifiable comparison independent of reactor type and process, we compare the methane conversion rate and the hydrogen production rate normalized per gram of catalyst. CH_4_ conversion rate (r) comes to 4 and 2.5 per gram of catalyst, respectively, for the first MP and after PT pyrolysis, still indicating a decrease in activity. This drop in performance might be attributed to the oxidized surface observed by XPS, which seems to require an in situ reduction phase by the methane feed before reaching optimal activity. On the other hand, comparing H_2_ production rates, as seen in Figure 5b, we see a slight increase in activity from 14 mmol/g/h to around 20 mmol/g/h after PT, shown in Figure 5c. The residual gas composition (Figure 5d) of N_2_ and Ar comes from the initial CH_4_ content and measurement itself, as some amount of atmosphere is left in the connections, while CO and CO_2_ are the products of the reaction. It is noteworthy that the amount of these residuals is rather small: N_2_ is under 1% while CO reaches 2.5%. It is assumed that CO comes from the reduction of initial iron oxide. Changes in production rates indicate increased selectivity; it is noteworthy that some amount of soot deposition happens on the reactor walls. This is attributed to partial carbon deposition via direct cracking of methane and carbon detachment. As the normalized carbon amount for calculations is based on carbon deposited on the catalyst, the increased hydrogen reaction rate is lower than estimated here. Nonetheless, this indicates that even though PT at these experimental conditions does not provide substantial surface cleaning, with partial Fe redeposition, it introduced surface defects that lead to comparable reaction rates. Further optimization is needed to achieve feasible regeneration and provide proof of concept that PT can aid catalyst regeneration.

## 3. Materials and Methods

### 3.1. Primary Material

The primary (fresh) catalyst consists of γ-Al_2_O_3_ as a catalyst support, Fe as a base catalyst material, and Ni nanoclusters deposited on top of Fe, helping to initiate the methane pyrolysis at lower temperatures comparing with a pure Fe catalyst. The material consists of about 54 at. % O, 24 at. % C, 13 at. % Al, 6 at. % Fe, and 2 at. % Ni. More information about the primary catalyst material can be found in our previous work [57]. The catalyst powders were analyzed using scanning electron microscopy (SEM) (Verios 4 G HP, Thermo Fisher Scientific, Waltham, MA, USA, and JSM 7600F, Jeol, Japan, equipped with EDXS Oxford Instruments, Abingdon, UK). Two types of samples were prepared for analysis. First, catalyst powders were mounted directly onto conductive carbon tape. Second, polished samples were prepared by mixing the catalyst powder with epoxy resin. After polymerization, the resulting compacts were polished using standard metallographic techniques. Prior to the analyses, the samples were coated with carbon to provide electrical conductivity and to prevent charging effects. The secondary-electron images were collected at 5 kV. The backscattering images were collected at 15 kV.

Figure S3a in the Supplementary Materials shows an SEM image of the powder, indicating that it is agglomerated. Figure S3b,c in the Supplementary Materials represent cross-sectional backscattered SEM images of an agglomerate. Micrometer-sized particles (gray phase, GP) are clearly visible and are coated with a thin layer of a brighter phase (BP). EDXS analysis (Figure S3d in the Supplementary Materials) of the GP revealed the presence of Al and oxygen, whereas Fe and Ni were additionally detected in the BP. These results confirm that the γ-Al_2_O_3_ powder is homogeneously coated with Fe and Ni.

### 3.2. Methane Pyrolysis and the Coked Catalyst

The primary material was employed as a catalyst for hydrogen production via methane pyrolysis. MP was carried out using an in-house-built reactor. A schematic representation has been shown in our previous work [57]. Shortly, the catalyst was placed in the chamber, which was evacuated with a fore vacuum pump, and then methane was introduced into the reactor at room temperature. Methane is filled to 0.5 bar, and then pyrolysis is carried out in three stages: first, ramping to a selected temperature over 120 min; second, holding the reaction temperature for 240 min; and third, natural cooling to room temperature. Reaction temperatures of the pristine catalyst were 700 °C, 800 °C, and 900 °C, respectively indicated as P700, P800, and P900, while the regenerated catalysts’ reaction temperature was set to 700 °C for all regenerated samples; thus, the secondary MP sample results are indicated as R(P700), R(P800), and R(P900). In every experiment, two gas samples were taken: first, a reactor gaseous sample before the start of the reaction; second, reactor gaseous content after cooling to room temperature, thus, ensuring clear view of the changes and CH_4_ conversion. Gas samples are collected using Swagelok sampling tanks with VCR connection, and then tested using mass spectrometry (RGA100 MS, Stanford Research Systems, Sunnyvale, CA, USA). Content is estimated based on the relative peak intensities and assumption of constant intensity ratios within the MS device and are assigned accordingly. The device is calibrated using industrial gas mixtures and CO from Linde gas. Sample and thus gas content before and after the methane conversion rate is known can be evaluated as ratio of final vs. initial per mass unit of catalyst:

(1) $$r=\left(1-\frac{CH_{4}\left(fin\right)}{CH_{4}\left(initial\right)}\right)\cdot m^{-1}$$

The hydrogen production rate is calculated similarly: after normalization, we take produced hydrogen amount in mol per mass unit of catalyst, per reaction time, thus producing a reaction rate with dimensions of $mmol\cdot g^{-1}\cdot h^{-1}$. After the reaction, the spent catalyst, which was covered by carbon deposits (coke), was removed from the reactor and used in NTP treatment. The gas analysis results are shown in Figure 5 for CH_4_ and H_2_, while the presence and amounts of other gases are shown in Figure 5d.

### 3.3. Coke Removal via Plasma Treatment

**Figure 6:**
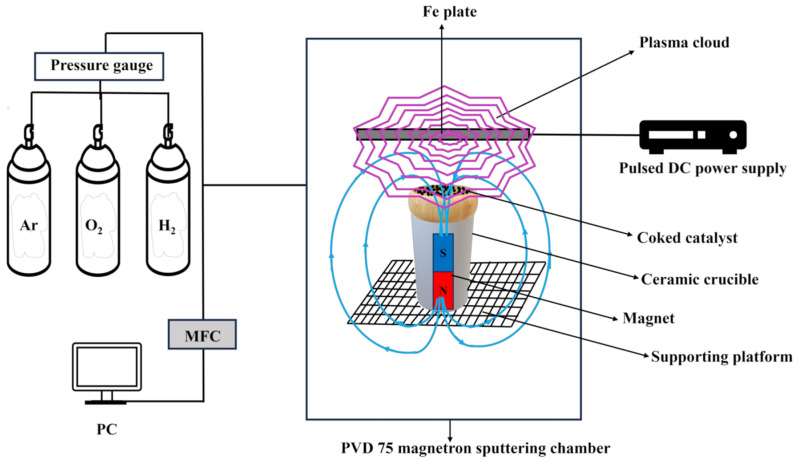
Schematic of the PVD 75 sputtering chamber for the assisted plasma treatment setup of deactivated catalyst. Pink lines represent plasma, while blue lines represent magnetic flow.

A magnetron physical vapor deposition (PVD 75) chamber fitted with a modified setup was used for the non-thermal plasma treatment of a deactivated catalyst. Catalyst regeneration was conducted in a modified PVD 75 chamber under high-vacuum conditions. The system is connected to three gas cylinders (H_2_, Ar, and O_2_) with rotary and cryogenic pumps employed to reduce the chamber pressure. The schematic representation of the entire system is shown in Figure 6. The in-chamber setup consists of a gas injection system (mentioned gas or their mixtures), an Fe target for plasma generation, a Petri dish, a ceramic crucible as a Petri dish holder, and a magnet inside the ceramic crucible to focus higher plasma intensity on the surface of the coked catalyst. An Fe target was controlled by the pulsed DC power supply (Advanced Energy Sparc-le 20 arc handling interface) to initiate a glow discharge plasma. The deactivated catalyst was loaded into a Petri dish to facilitate plasma-assisted coke removal. Before the plasma treatment, air was evacuated from the vacuum chamber to reach a high vacuum (≈10^−6^ mbar), after which the working gas was introduced to reach a pressure of 9 × 10^−2^ mbar pressure. The Petri dish containing coked catalyst powder was positioned below the Fe target; the distance between the Fe target and the catalyst was 20 mm, while the distance between the Fe target and the magnet was 30 mm. The cylindrical-shaped magnet parameters were as follows: height: 35 mm, diameter: 12 mm, axially magnetized with a surface field strength ≈ 1.2 T.

The main plasma treatment parameters were set as follows: the current was ≈0.6 A, the voltage was 700 V, and the total power was ≈400 W. The plasma treatment was carried out for 60 min, with one intermediate interruption after 30 min. During this break, the process was stopped, and the powder was removed from the vacuum chamber for manual mixing to improve plasma exposure of the powder surface. After mixing, the powder was returned to the vacuum chamber, and the treatment was continued under identical conditions. All plasma treatment parameters were selected based on preliminary optimization experiments.

### 3.4. Chemical and Structural Analysis

During plasma treatment, a small amount of Fe was deposited on top of the catalyst even under pure H_2_ conditions. Therefore, the deposited amount of Fe was evaluated by using Bruker DektakXT stylus profilometer. Pure quartz glass substrates were used instead of coke-catalyzed catalysts to maintain stable plasma treatment conditions. For these measurements, the quartz substrates were placed in the same position as the catalyst samples inside the plasma reactor and treated under identical plasma conditions. After treatment, the step height between the deposited region and the non-exposed reference area of the quartz substrate was measured by stylus profilometry and used to estimate the apparent Fe layer thickness reported in Table 1. These measurements were used only to assess the tendency for Fe re-deposition from the plasma target and to aid interpretation of the catalyst results; they do not represent direct quantification of Fe deposited on the porous catalyst surface itself, where deposition behavior may differ because of powder morphology.

The morphological examinations of the treated catalysts were performed using scanning electron microscopy (SEM, Hitachi S-3400N, Tokyo, Japan). Elemental composition was determined using energy-dispersive X-ray spectroscopy (EDS, Bruker Quad 5040, Hamburg, Germany). Structural analysis of the plasma-treated catalysts was performed using X-ray diffraction (XRD) on a Bruker D8 diffractometer operated at 40 kV and 40 mA in the θ–θ configuration. The diffraction pattern was obtained over a 2θ range of 20–70° utilizing Cu Kα radiation with λ = 0.15406 nm. Phase identification of the plasma-treated sample was analyzed using the PDF-5+ database (ICDD) as a reference. The surface elemental composition and chemical states of the treated catalyst were examined using X-ray photoelectron spectroscopy (XPS, PHI 5000 Versaprobe, Boston, MA, USA). Because XPS is inherently surface-sensitive, it was used here to evaluate changes in the outermost catalyst surface after plasma treatment, including changes in surface carbon concentration and Fe chemical state.

## 4. Conclusions

This study evaluated the potential of non-thermal plasma (NTP) as a regeneration concept for coked catalysts deactivated during methane pyrolysis. The results demonstrate that NTP treatment can effectively promote partial carbon removal from the catalyst surface through in situ plasma-assisted reactions. Under the investigated conditions, a maximum surface carbon removal of approximately 38% was achieved using a H_2_ (95%) and O_2_ (5%) gas mixture, 400 W (0.6 A), and a 1 h treatment time.

Characterization analyses using SEM, EDS, XPS, and XRD revealed that plasma treatment altered the surface composition and partially removed carbon deposits while maintaining the overall structural integrity of the catalyst. An auxiliary magnet was incorporated to guide the plasma flux toward the catalyst region, thereby focusing the plasma flow on the catalyst surface, suggesting that plasma directionality can influence regeneration efficiency. However, methane pyrolysis tests revealed that the achieved carbon removal was insufficient to substantially restore catalytic activity, with hydrogen production recovering to only 20% of the initial performance. At this stage, the method should therefore be regarded as a preliminary proof-of-concept rather than a practically effective regeneration route. These findings indicate that although NTP may offer a cleaner alternative to conventional regeneration methods, further optimization of plasma parameters, reactor configuration, and treatment conditions are necessary to improve carbon removal efficiency and catalytic activity recovery.

## Figures and Tables

**Table 1:** Changes in elemental concentrations by XPS after plasma treatment experiments.

Sample	Elemental Concentration, at. %				Surface Carbon Removal, %	Fe Thickness on Quartz, nm
	C	O	Al	Fe		
Coked catalyst	77.4	15.4	7.2	-		
H_2_ (100%)	76.2	16.5	6.7	0.6	1.5	>50
H_2_ (97%) + Ar (3%)	71.4	18.9	8.7	1.0	7.8	80
H_2_ (95%) + Ar (5%)	64.1	24.3	9.6	2.0	17.1	135
H_2_ (90%) + Ar (10%)	56.3	31.3	6.3	6.1	27.3	250
H_2_ (80%) + Ar (20%)	51.7	35.2	5.5	7.6	33.2	300
H_2_ (97%) + O_2_ (3%)	67.7	22.3	8.2	1.8	12.5	>50
H_2_ (95%) + O_2_ (5%)	49.8	35.3	11.3	3.6	35.6	61
H_2_ (90%) + O_2_ (10%)	49.6	35.6	11.0	3.8	35.9	103
H_2_ (80%) + O_2_ (20%)	47.5	37.7	8.0	6.8	38.6	160

## Data Availability

Data are contained within this article.

## Supplementary Materials

The following supporting information can be downloaded at: https://www.mdpi.com/article/10.3390/molecules31101733/s1, Figure S1. XPS survey spectra of the catalyst before and after plasma treatment by H_2_-O_2_ gas mixture; Figure S2. EDS elemental map of the coked catalyst (a) before and (b) after regeneration; Figure S3. SEM images of (a) the catalyst mounted on carbon tape, (b) and (c) the polished sample, and (d) EDXS spectra of phases, detected in the primary material.
