# Supplementary material for: Investigation of Coked Catalyst Regeneration via Non-Thermal Plasma Treatment and Its Reuse for Hydrogen Production from Methane Pyrolysis
Source: Molecules. 2026 May 19;31(10):1733. doi: 10.3390/molecules31101733 (PMC13209207; doi:10.3390/molecules31101733)
Supplement: Supplementary file 1 [file molecules-31-01733-s001.zip › molecules-4233247-supplementary (1).pdf]

Supplementary Materials

Supplementary Material of the article "Investigation of the coked catalyst regeneration via non-thermal plasma treatment and its reuse for hydrogen production from methane pyrolysis" in the Journal Molecules MDPI.

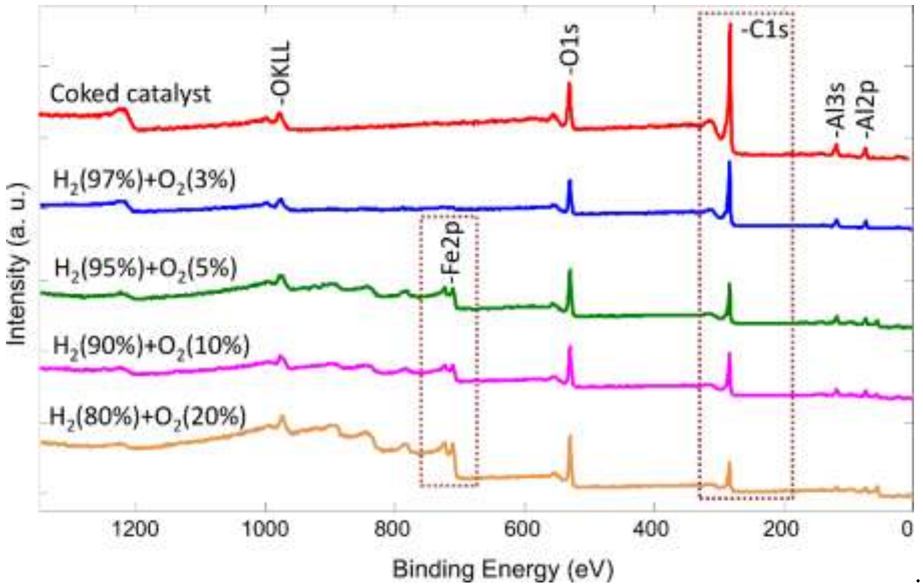

Figure S1. XPS survey spectra of the catalyst before and after plasma treatment by H_2-O_2 gas mixture.

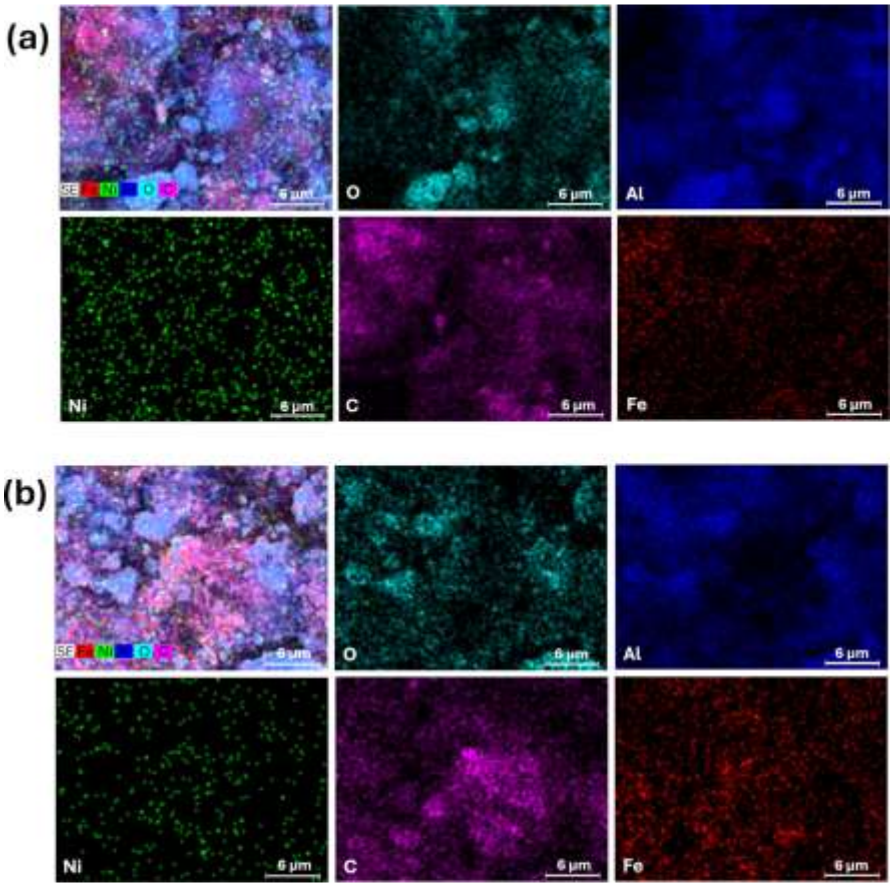

Figure S2. EDS elemental map of the coked catalyst (a) before and (b) after regeneration.

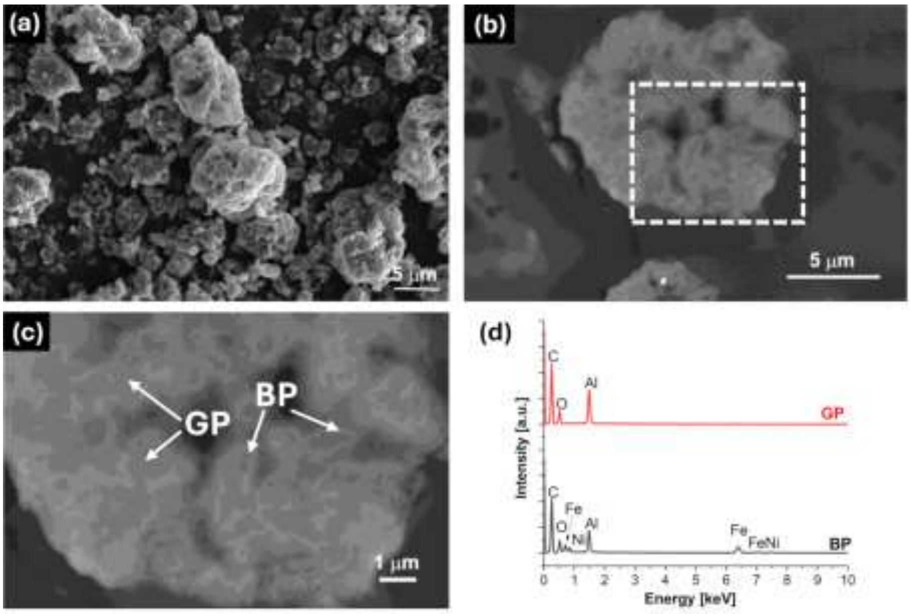

Figure S3. SEM images of (a) the catalyst mounted on carbon tape, (b) and (c) the polished sample, and (d) EDXS spectra of phases, detected in the primary material.
